# Quantum–Quantum and Quantum–Quantum-Classical Schemes for Near-Gap Excitations with Projection-Based-Embedded *GW*-Bethe–Salpeter Equation

**DOI:** 10.1021/acs.jctc.4c00163

**Published:** 2024-06-25

**Authors:** Vivek Sundaram, Björn Baumeier

**Affiliations:** † Department of Mathematics and Computer Science, Eindhoven University of Technology, P.O. Box 513, 5600MB Eindhoven, The Netherlands; ‡ Institute for Complex Molecular Systems, Eindhoven University of Technology, P.O. Box 513, 5600MB Eindhoven, The Netherlands; ¶ Department of Applied Physics and Science Education, Eindhoven University of Technology, P.O. Box 513, 5600MB Eindhoven, The Netherlands

## Abstract

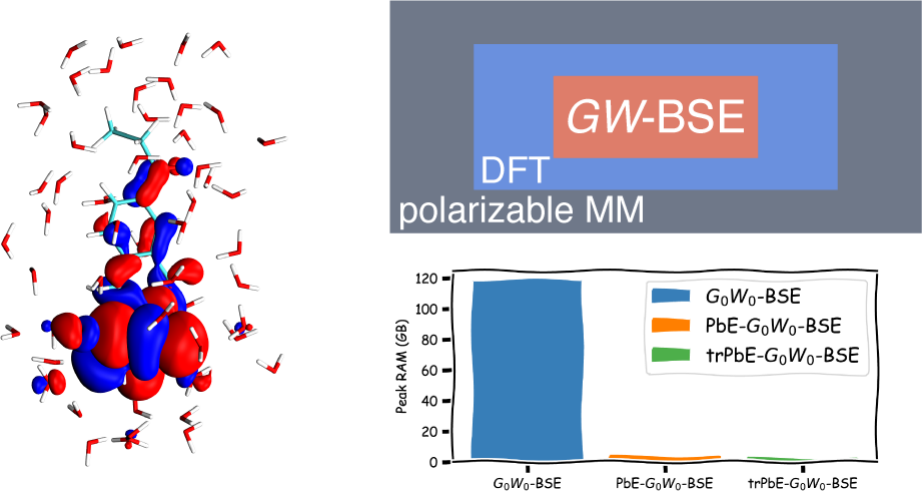

We present quantum–quantum and quantum–quantum-classical schemes based on many-body Green’s functions theory in the *GW* approximation with the Bethe–Salpeter equation (*GW*-BSE) employing projection-based-embedding (PbE). Such approaches allow defining active and inactive subsystems of larger, complex molecular systems, with only the smaller active subsystem being explicitly treated by *GW*-BSE offering significant computational advantages. However, as PbE can modify the single-particle states in the Kohn–Sham (KS) ground state calculation and screening effects from the inactive region are not automatically included in *GW*-BSE, results from such PbE-*GW*-BSE calculations can deviate from a full-system reference. Here, we scrutinize in detail, e.g., the individual and combined effects of different choices of active regions, the influence of omitting the screening from the inactive region, and strategies for basis set truncation on frontier orbital and near-gap electron–hole excitation energies. As prototypical systems, we consider a diketopyrrolopyrrole bicyclic ring including side-chains, a polarity-sensitive dye (prodan) in aqueous environment, and a π-stacked dimer of benzene and tetracyanoethylene in water, respectively, covering a variety of excitation characters in molecular systems with complex chemical environments and photoinduced processes. Our results suggest that to obtain agreement of approximately 0.1 eV between near-gap excitation energies from embedded and full calculations, the active region should be chosen based on the Mulliken population of the full highest-occupied molecular orbital and that careful benchmarking should be done on the KS level before the actual *GW*-BSE steps when basis set truncation is used. We find that PbE-*GW*-BSE offers significant reductions in computation times and, more importantly, memory requirements, making calculations for considerably larger systems tractable.

## 1 Introduction

Many-body Green’s functions Theory employing the *GW* approximation and the Bethe–Salpeter equation (BSE)^1^ has been a widely established method for the determination of electronic excitations in solid-state physics. Over the past decade, it has gradually found more and more application in traditionally molecular quantum chemistry settings.^2−10^ It was shown that *GW*-BSE provides an effective single- and two-particle picture with accurate energies for charged and neutral excitations of different character, e.g., photoionization and localized vs charge-transfer type excitations, without the need for any adaptations.^4,11,12^ Even though its scaling (dependent on details of the implementation) is favorable compared to wave function based methods such as ADC(2)^13^ or CC2,^14^ the direct application of *GW*-BSE to many complex molecular systems remains computationally challenging. Examples of such molecular systems are polymers with complex internal architecture, either solvated^15^ or pure or mixed blends,^16^ more general solvent–solute systems with nonequilibrium relaxation dynamics,^17^ or molecular aggregates as in organic semiconductor films.^18^

To make systems like these accessible, hybrid methods combining quantum and classical methods are often used,^4,17,19−27^ sometimes combined with machine-learning models.^28^ While effective, such approaches rely on, e.g., some intuitive partitioning of the supramolecular system into fragments that only interact via classical electrostatics and a careful choice and parametrization of the environment model, and may fail for covalently or hydrogen-bonded systems with partial charge transfer. In these cases, a quantum–quantum embedding approach might be advantageous, which allows defining active and inactive partitions, with only the smaller active partition being explicitly treated by *GW*-BSE, while interaction with the inactive one is on the level of density-functional theory (DFT).^29−31^ Recently, Tölle et al.^32,33^ have reported *GW*-BSE calculations based on subsystem-DFT (sDFT)^34−38^ for a series of weakly interacting molecular clusters. sDFT starts from Kohn–Sham-like calculations on fragments and determines the full-system density from them, in which the effective potential for a fragment contains contributions from nonadditive terms in the kinetic energy and exchange-correlation potential. This intrinsic partitioning of the supramolecular system into small fragments allowed the authors to also partition the screening contributions to the correlation part of the self-energy in *GW* and to approximately include environment polarization effects into the calculations, and it could be shown to recover to a large extent the full-system frontier orbital energies and the transitions between them.

Projection-based-embedding^39−44^ (PbE) is an alternative to sDFT, which partitions the full system based on a full-system reference Kohn–Sham calculation into active and inactive parts, and to subsequently restrict the *GW*-BSE calculation to the active part. PbE has some advantages over sDFT, e.g., it is a formally exact partitioning and also works for active/inactive partitioning through covalent bonds, which is important for studies of macromolecular assemblies with electronically active vs inactive functional groups. However, for large inactive parts involving many molecules or molecular fragments adopting the environment screening correction proposed in^32^ becomes cumbersome, as it would require either a screening calculation for a still intractably large single inactive region or further decomposition of the latter.

**Figure 1:**
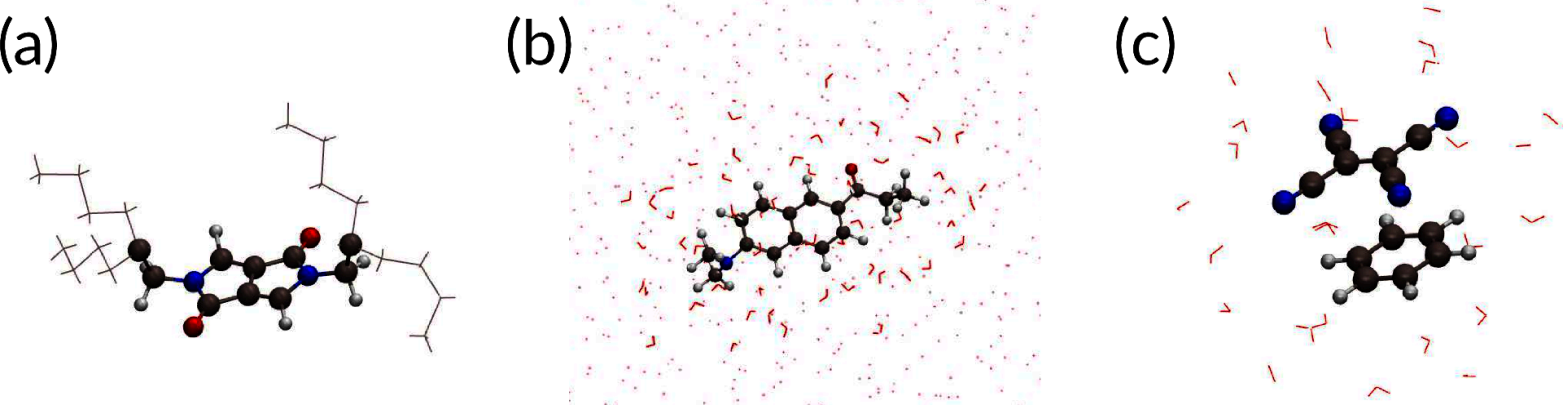
Molecular structures used as test systems for PbE-*GW*-BSE: (a) DPP bicyclic ring with branched alkyl side chains, (b) prodan in close (quantum) and distant (classical) water, (c) a water-solvated benzene-TCNE dimer.

Arguably there are scenarios in which the exact agreement between a (hypothetical) full supramolecular and a PbE-*GW*-BSE calculation is not required. For dynamical processes such as charge or exciton transfer or conversions between localized and charge-transfer type excitations, for instance, only relative energy gaps are relevant. Against this background, we scrutinize in this work in detail different schemes of PbE-*GW*-BSE calculations: plain calculations, calculations in which a truncated atomic orbital basis is used, and calculations in which the PbE-*GW*-BSE calculation is further embedded in a classical, atomistic polarizable region. We aim to elucidate the individual and combined effects of different choices of active regions, the influence of screening (or lack thereof) from the inactive region, and the impact of basis set truncation on energies of local and charge-transfer excitations, respectively. As prototypical systems covering different types of excitations in a variety of chemical environments, we consider the three test systems as shown in Figure 1: (a) a single diketopyrrolopyrrole (DPP) bicyclic ring with branched alkyl side-chains, (b) prodan, a polarity-sensitive dye, solvated in water, and (c) a benenze-TCNE donor–acceptor pair in water. For all systems, we study the effects of the PbE with (trPbE) or without basis truncation on the frontier orbitals as well as the selected local or CT excitations. We pay special attention to the differences in contributions of the exchange and correlation parts of the self-energy to quasiparticle energies and of exchange and direct terms in the electron–hole interaction to the BSE energies between full and embedded calculations. In addition to this, each of the three test systems is here chosen with specific objectives: For the DPP molecule with branched side chains, we intend to demonstrate a PbE-*GW*-BSE calculation for a system in which the two regions are connected by a covalent bond. We investigate the sensitivity of the embedding results on the choice of the active region. Prodan in water has been chosen to showcase the quantum–quantum-classical PbE-*GW*-BSE/MM approach. Also here, we consider the influence of the choice of the active regions on the predicted excitation energies. The donor–acceptor benzene-TCNE dimer solvated in water is used to evaluate the differences in embedding effects on localized and charge-transfer type excitations.

This paper is organized as follows: Section 2 summarizes the essentials of many-body Green’s functions theory, projection-based-embedding and basis set truncation methods, as well as the coupling to classical polarizable environments. Computational details are given in Section 3 before the results for the three test systems are presented in Section 4 and the overall findings are discussed in Section 5. A brief summary concludes the paper.

## 2 Methodology

This Section provides a concise overview of the essentials of the different methodologies used in this work. For detailed discussions, e.g., of recommended numerical parameters, we refer to the appropriate original literature at the respective places.

### 2.1 Many-Body Green’s Functions Methods for Electronically Excited States

Kohn–Sham (KS) DFT^45,46^ provides the starting point for the effective single- or two-particle formulations for electronic excitations and their energies within the framework of perturbation theory with many-body Green’s functions. One first obtains KS wave functions ϕ_*i*_^KS^(**r**) and energies ε_*i*_^KS^ from
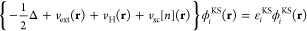
1where *v*_ext_ is the external potential, *v*_H_ the Hartree potential, and *v*_xc_ the exchange-correlation potential, and they define together with the kinetic energy operator the effective KS Hamiltonian *Ĥ*_KS_.

Hedin^47,48^ introduced the *GW* approximation of many-body Green’s functions theory, in which electron self-energy is written as Σ = *iGW*, and allows to derive a set of effective single-particle eigenvalue problems known as the *quasiparticle* (QP) equations
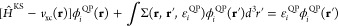
2Typically, the QP wave functions ϕ_*i*_^QP^(**r**) are approximated by the KS wave functions, which allows to write the QP energies as
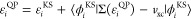
3The self-energy is calculated in frequency space (with η → 0^+^ to ensure convergence) as
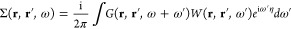
4from the Green’s function based on the Kohn–Sham solution
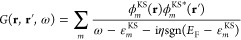
5and the screened Coulomb interaction *W* in the random-phase approximation
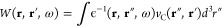
6Evaluating eq 6 in turn requires the microscopic, frequency-dependent dielectric function given by
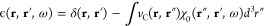
7containing the irreducible polarizability χ_0_:
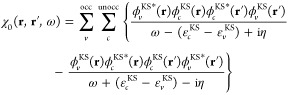
8As the self-energy is energy-dependent, and thus depends on ε_*i*_^QP^, the solution of eq 3 must be found self-consistently. From eq 7 it is possible to split the self-energy Σ = i*GW* into its bare exchange part
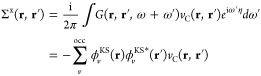
9and the explicitly frequency-dependent correlation part
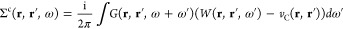
10

With ω_*i*_ = ε_*i*_^KS^ + ⟨ϕ_*i*_^KS^|Σ^x^ – *v*_xc_|ϕ_*i*_^KS^⟩ and ⟨ϕ_*i*_^KS^| Σ^c^(ω)|ϕ_*i*_^KS^⟩ = Σ_*i*_^c^(ω), we can rewrite eq 3 into the fixed-point problem
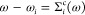
11Due to the pole structure of the self-energy, there are in general several solutions to eq 11. In this situation, the spectral weight, defined as
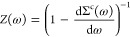
12is used to identify the ”true” QP energy by *Z*(ω) ≈ 1, or |d Σ^c^(ω)/dω| ≈ 0.

In the above, when evaluating the self-energy, the KS eigenvalues and eigenfunctions are used to construct *G* and *W*, which is also known as a ”one-shot” *G*_0_*W*_0_ calculation. Alternatively, it is possible to use updated QP energies until eigenvalue self-consistency is reached (ev*GW*).^49−51^

Charge-neutral excitations that involve excitonic effects (electron–hole pair interaction) are not accounted for, and can instead to obtained so solutions to the *Bethe–Salpeter equation* (BSE)
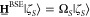
13in which the electron–hole wave functions |ζ_*S*_⟩ are typically expressed in a basis of resonant and antiresonant products of single-particle functions
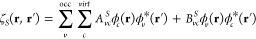
14With that, the BSE explicitly reads in matrix form
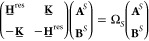
15with elements
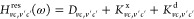
16
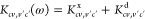
17and
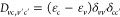
18
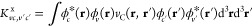
19
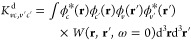
20If the ground state is a spin-singlet state and spin–orbit coupling is small compared to the electron–hole coupling, the BSE solutions can be classified as spin-singlet and spin-triplet excitations. This allows in turn, to solve the BSE separately for the spin type of interest, specifically for singlet excitations with
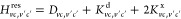
21
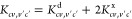
22

### 2.2 Projection-Based Embedding

Projection-based embedding (PbE) is a formally exact DFT-in-DFT embedding scheme, which we briefly summarize here and refer the reader to the original work by Manby et al.^39^ for full technical details. The scheme begins with a standard DFT calculation on the complete reference system in the full-molecule AO basis. The *N* occupied canonical molecular orbitals from this calculation, ϕ_*i*_(**r**) for *i* = 1, ... *N*, are then first transformed into localized orbitals, ϕ_*i*_^LO^(**r**), with a unitary transformation that leaves the total density of the system unchanged. With the specification of a set of atoms in the active region A, one constructs an initial active density *n*^A^(**r**) from those localized orbitals that have a significant Mulliken population *q*_*i*_^A^ > *q*_*t*_ on these atoms (*q*_*t*_ is typically 0.4 as recommended from in ref (39).):
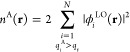
23With *n*^A^(**r**) given, one can determine the density of the inactive region as *n*^B^(**r**) = *n*(**r**) – *n*^A^(**r**). From this initial partitioning of the total reference density, one considers *n*^A^(**r**) variable, denoted here as *ñ*^A^(**r**). The Fock matrix in the full-molecule AO basis for an embedded (A-in-B) calculation on the electrons in subsystem A is given by
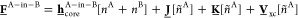
24where **h**_core_^A-in-B^ is the embedded core Hamiltonian based on the partitioned initial densities *n*^A^ and *n*^B^. The density-dependent terms **J**, **K**, and **V**_xc_ are updated in each iterative step and hence depend on the updated active density *ñ*^A^. The embedded core Hamiltonian reads
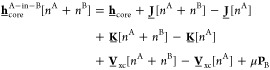
25and contains the core Hamiltonian of the full system, the difference between the Hartree, exchange, and exchange-correlation terms for the full system and the initially chosen active subsystem, respectively, as well as a projection term μ**P**_B_ with the projection operator
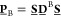
26based on the density matrix **D**^B^ of the environment and the atomic orbital overlap matrix **S** to ensure orthogonality between the occupied states of the environment and the rest of the active subsystem. In the limit μ → ∞, the two subsystems are exactly orthogonal. In practical calculations, we adopt a value of μ = 10^5^ Hartree, based on our own convergence tests and those performed in ref (39).

Performing a PbE calculation allows us to limit a *GW*-BSE calculation for the electronic excitations on the active region. As the embedding potential is already included in the preceding PbE-DFT calculation, there are no changes to the procedure of the *GW*-BSE steps as outlined in Section 2.1, except that all quantities involved use the embedded Kohn–Sham molecular orbitals and their energies as starting point. We will discuss the impact on the results of such calculations in Section 4.

### 2.3 Truncation of the Full Atomic Orbital Basis

All matrices composing the Fock matrix **F**^A-in-B^ are so far expressed in the full molecular atomic orbital basis. The most noteworthy computational gain of the embedding lies in the fact that only a smaller number of occupied states need to be explicitly determined in the self-consistent procedure.

Reducing the atomic orbital basis for the actual embedding step should not only offer computational savings by decreasing the dimension of the eigensystem, but also affect the virtual orbital space and, in an extreme case, localize the orbitals intrinsically in the active region.

Miller et al. have shown that a reduced atomic orbital basis can be constructed by truncating the full basis of the reference calculation via manipulation of the projection term.^43,44^ The procedure begins with an additional classification of atoms in the inactive region into *border atoms* and *distant atoms*. This distinction is based on whether any of the atomic basis functions centered at an atom of the inactive region contribute beyond a threshold to the density of the active region. In practice, this is determined based on the net Mulliken population of an atomic orbital α in the active density matrix **D**^A^
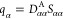
27where **S** is the overlap matrix. If any of the *q*_α_ exceeds a threshold value (typically 10^–4^ as taken from refs (43 and 44)), the atom associated with basis function α is added to the list of border atoms. All remaining atoms are distant atoms. Subsequently, the originally assigned inactive molecular orbitals are also split into border and distant MOs. Border MOs are inactive molecular orbitals that have a Mulliken population larger than a threshold (a value of 0.4 is suggested in refs (43 and 44)) on any of the border atoms. All remaining MOs are distant molecular orbitals.

This splitting into border and distant molecular orbitals also allows a similar splitting of the projection operator via the respective density matrices **D**^border^ and **D**^distant^
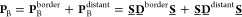
28The split projector is now used in eq 25, however, with different values for the level-shift, such that μ**P**_B_ ⇒μ^border^**P**_B_^border^ + μ^distant^**P**_B_^distant^. However, it was shown by Barnes et al.^43^ that enforcing orthogonality between the active subsystem in the truncated basis and the inactive MOs outside of this basis can lead to significant numerical errors. They authors showed that this problem can be avoided by using μ^distant^ = 0 and found this way good accuracy between the total energies in trPBE and full calculations with hardly any dependence on the value of μ^border^ in the range of 10^2^ – 10^6^ Hartree. These steps allow evaluating the Fock matrix in eq 24 in a reduced, truncated, basis which only includes the basis functions centered at the active and border atoms. We refer to this as truncated projection-based-embedding (trPbE), in the remainder.

### 2.4 Classical Polarizable Embedding

To account for the effects of a complex molecular environment on electronic excitations, a quantum (QM) region with the excited state complex is embedded in a classical, polarizable atomistic (MM) model for the environment. The QM/MM scheme in VOTCA-XTP makes use of a distributed atomic multipole representation for molecules in the MM region, which allows treatment of both the effects of static electric fields and the polarization response as a self-consistent reaction field. Specifically, this classical MM energy for the system is evaluated as
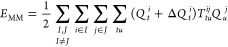
29where *I* and *J* indicate individual molecules in the system, *i* and *j* atoms in the respective molecules, *Q*_*t*_^*i*^ are the static atomic multipole moments of rank *t* associated with atom *i*, and *T*_*tu*_^*ij*^ is the tensor describing the interactions between the multipoles moments *Q*_*t*_^*i*^ and *Q*_*u*_^*j*^.^52^ The induced moments Δ*Q*_*t*_^*i*^ are generated by the electric field created by moments *t*′ of atom *i*′ ≠*i* in molecule *I* and the one generated by the moment *u* of atom *j* in molecule *J*:
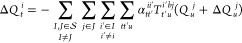
30with α_*tt*′_^*ii*′^ the atomic polarizability on each site. To avoid the effects of spurious overpolarization, a damped version of the interaction tensor (Thole damping^52^) is used. Then, the static and induced multipoles in the MM region also interact with the electron density in the QM region via an additional external potential to eq 1. At the same time, the explicit electrostatic field from the QM density is included in polarizing the MM region. The total density of excited state *S* is evaluated from the excited-state wave function ζ^*S*^ as
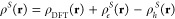
31with
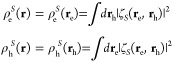
32

To obtain the polarization response of both the QM and MM regions, a self-consistent procedure is employed. At step *p* of this procedure, the total energy of the coupled QM/MM system for the state *S* of interest (ground state *S* = 0, or excited states *S* > 0) is determined as
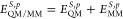
33with
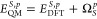
34and Ω^*p*^_*S*_ = 0 for the ground state case. The whole procedure is repeated until the change of total energy is less than a preselected accuracy, typically 10^–5^ Ha. The excitation energy Ω_*S*_^QM/*MM*^ of a complex in the polarizable environment is then obtained as the difference
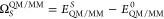
35

As in this the interactions between the quantum and classical regions are purely represented by electrostatic potentials, it is straightforward to combine (tr)PbE and classical polarizable embedding approaches into one quantum–quantum-classical embedding scheme. The static moments of the MM regions continue acting as an additional background potential to the quantum–quantum region. Similarly, the electric field acting on the polarizable sites in the MM region is created by the total electron density (plus the nuclei) of the subsystem QM region. It is worth highlighting that when a polarizable model is used in the PbE-*GW*-BSE/MM, the outer SCF coupling the quantum and classical regions implies that during such a calculation also the density of the inactive region can respond to the polarization of the MM region.

## 3 Computational Details

All calculations have been done using the VOTCA-XTP package^22,23^ which interface to the ORCA software^53^ for the full system reference DFT calculations. The def2-TZVP basis-set^54^ with an optimized auxiliary basis^55^ along with the PBE0 hybrid functional^56^ has been used in all DFT and subsequent *GW*-BSE calculations. For the construction of localized orbitals required in the projection-based-embedding calculations, we employ the Pipek–Mezey (PM) localization scheme,^57^ which maximizes the atomic Mulliken population subject to the constraint of keeping the total density fixed. For the actual maximization step, we make use of the unitary optimization algorithm as described by Lehtola and Jonsson.^58^ If not stated otherwise, the *G*_0_*W*_0_ variant has been chosen with the Plasmon-Pole model^59^ (PPM) for self-energy calculation in the *GW* step. To maintain consistency, we use in each case the full spectrum of single-particle states in the RPA (*N*_RPA_), and consider all occupied (*N*_occ_) and the lowest *N*_virt_ = 2*N*_occ_–1 unoccupied states for the calculation of the QP corrections and the expansion of the BSE product basis, i.e., *N*_BSE_ = 2*N*_occ_(2*N*_occ_–1) . The explicit numbers for all three test systems are summarized in Table 1.

**Table 1:** Overview of Number of Basis Functions (*N*_basis_), Number of Functions in Auxiliary Basis (*N*_aux_), Number of Occupied States Included in Quasiparticle Calculation and BSE Product Basis (*N*_occ_), Idem for Virtual States (*N*_virt_), Number of Transitions in RPA (*N*_RPA_), and Dimension of BSE Hamiltonian (*N*_BSE_), for Full-*GW*-BSE, PbE-*GW*-BSE, and trPbE-*GW*-BSE Calculations on the Three Test Systems

	*N*_basis_	*N*_aux_	*N*_occ_	*N*_virt_	*N*_RPA_	*N*_BSE_
**DPP+alkyl**						
full-*GW*-BSE	1194	2940	115	229	124085	52670
PbE-*GW*-BSE	1194	2940	51	101	55029	10302
trPbE-*GW*-BSE	1152	2835	51	101	52887	10302
**aqueous prodan**						
full-*GW*-BSE	2994	7377	336	672	893088	451584
PbE-*GW*-BSE(dye)	2994	7377	61	121	162138	14762
PbE-*GW*-BSE	2994	7377	96	191	255168	36672
trPbE-*GW*-BSE(dye)	2453	6035	61	121	129137	14762
**aqueous benzene-TCNE**						
full-*GW*-BSE	1650	4062	183	365	268461	133590
PbE-*GW*-BSE	1650	4062	53	105	77751	11130
trPbE-*GW*-BSE(10^–4^)	1384	3400	53	105	63653	11130
trPbE-*GW*-BSE(10^–5^)	1638	4032	53	105	77115	11130

## 4 Results

### 4.1 General Considerations for Projector-Based-Embedded *GW*-BSE Calculations

While the DFT-in-DFT calculation can be shown to reproduce the full reference total energy exactly, we have seen that there are changes in the molecular orbitals, and we therefore cannot in general expect a *GW*-BSE calculation after PbE-DFT (from now on referred to for short as PbE-*GW*-BSE) to yield the same excitation energies as a full *GW*-BSE calculation. One can get an indication of what the general changes are by considering, e.g., the expressions for the exchange part Σ^x^ (eq 9) and correlation part Σ^c^ (eq 10) of the self-energy.

The exchange part is affected by (i) summing over fewer occupied states in the subsystem-*GW*-BSE calculation and (ii) the changes in the molecular orbitals themselves. Note that while Σ^x^ itself only depends on the occupied orbitals, it enters the quasiparticle energies of both occupied and unoccupied states as evaluated by eq 3. Therefore, even though the virtual molecular orbitals are unchanged in the subsystem-DFT calculation (using the full basis), their *GW* quasiparticle energies may have different contributions arising from Σ^x^.

For the frequency-dependent correlation part, a similar analysis is more complicated, as the expression in eq 10 involves the single-electron Green’s function (eq 5), and the screened Coulomb interaction *W* determined with the help of the irreducible polarizability χ_0_ as in eq 8. Subsystem embedding changes both *G*_1_ (which also leads to the discussed changes in Σ^x^) and χ_0_ via the different orbitals and their energies. For polarizability, embedding implies several noteworthy modifications. Even if the ϕ(**r**) and energies ε were unchanged, the sum over occupied orbitals is limited to the active occupied orbitals (the ones from the inactive one are found in the virtual space at high energy, and should be excluded from the sum over virtual orbitals). As a result, the screening only has contributions from transitions between occupied orbitals in the active subsystem and virtual orbitals of the combined system (in the full basis calculation), while contributions from transitions from occupied orbitals in the inactive region to all virtual orbitals are removed. The inactive region therefore can be considered static from the perspective of the screened Coulomb interaction, similar to a QM/MM embedding with only static moments in the MM region, as discussed in Section 2.4. If additionally a truncated basis restricted ideally to the active region is used, this will also affect the virtual orbitals and essentially limit the transitions to those within the active region, removing charge-transfer-like transitions between the subsystems from the response. The effect on the calculated contribution of these CT-like transitions to Σ^c^ is expected to be small, however. Both considerations regarding the modifications type of transitions excluded in the screening in subsystem-*GW* are in general then combined with additional effects of changed orbitals and their energies in the active region. From the lack of screening from the now inactive region, one can generally expect the contributions of Σ^c^ to the quasiparticle energies to be smaller (in absolute values) in the subsystem-*GW* calculation compared to the full *GW* case. In other words, even when the orbitals themselves are only minimally affected (for weakly interacting, nonbonded molecular structures, for instance), one can expect to find the occupied (virtual) quasiparticle energies from the embedded calculation to be below (above) the ones from the full calculations. In particular, the HOMO–LUMO gap in subsystem-*GW* is then larger than the respective gap in full-system *GW*.

One can make similar examinations on the level of the BSE. Naturally, as the number of occupied orbitals is reduced, the electron–hole transitions used to expand the two-particle wave functions are limited to the transitions starting from the active subsystem. Any changes to the quasiparticle energies as a result of the points discussed above will directly impact the free transition term *D*_*vc*,*v*′*c*′_ from eq 18 in the BSE Hamiltonian. The effects on the exchange and direct (screened) terms of the electron–hole interaction kernel *K*_*vc*,*v*′*c*′_^x^ and *K*_*vc*,*v*′*c*′_^d^ are similar to those discussed for Σ^x^ and Σ^c^, respectively. Especially, the reduced screening can be expected to result in stronger electron–hole attraction compared to the full-system calculation and might in turn compensate to some degree the larger quasiparticle gap in the free transition.

### 4.2 DPP Bicyclic Ring with Branched Alkyl Side-Chains

**Figure 2:**
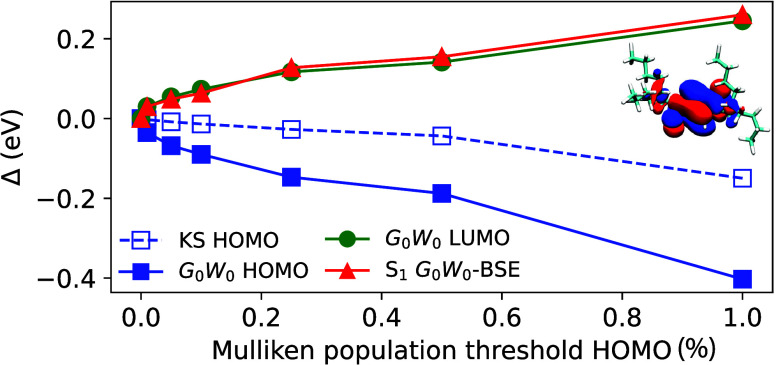
Deviation Δ (in eV) of calculated KS HOMO, *G*_0_*W*_0_ HOMO, *G*_0_*W*_0_ LUMO, and *G*_0_*W*_0_-BSE S_1_ energies between full and PbE calculations as a function of the Mulliken population threshold for the full KS HOMO used for including atoms in the active region. Inset shows isosurfaces of the KS HOMO (isovalues ±0.01 a_B_^–3^) of the single DPP bicyclic ring with branch alkyl side chain as obtained from regular KS-DFT.

As a first test system, we consider a single DPP unit. Alkyl side chains with a branched structure are attached to the respective nitrogen atoms. A short C_2_H_2_ group contained the branching point, and each branch is formed by C_4_H_9_, as can also be seen in Figure 1(a). The geometry of this structure is cut from a snapshot of a large-scale classical Molecular Dynamics simulation of a DPP2Py*m*T polymer^16^ and then relaxed in vacuum (DFT with the PBE0 functional and def2-TZVP basis) to a local minimum with nonsymmetric arrangement of the side chains. In (conjugated) polymer systems, it is often assumed that the frontier orbitals relevant for charge transport are localized on the actual functional backbone and that the side chains do not participate in the electronic processes. For the testing of the PbE-*GW*-BSE approach, such chemical intuition suggests actually selecting only the DPP unit including the nitrogen atoms as the active region and the complete two branched alkyl side chains into the inactive one. However, as can be seen from the isosurfaces of the HOMO from a full KS calculation in the inset of Figure 2, the occupied frontier orbital extends further into the side chains, even slightly beyond the branching atom. The choice of only the DPP core as the active region is therefore expected to yield considerable modifications to the occupied electronic states.

To understand how the addition of side-chain atoms influence the results of PbE calculations on the near-gap excitations, we vary their number based on the Mulliken population of the atoms in the full KS HOMO depending on a threshold value. If the population exceeds this threshold, we add the atom to the active region. We chose the KS HOMO population because (i) the LUMO is unaffected in PbE as discussed above and (ii) we are interested in the near-gap electronic structure. Figure 2 shows the difference Δ between the results for the KS HOMO, the *G*_0_*W*_0_ HOMO and LUMO, and the S_1_*G*_0_*W*_0_-BSE energies obtained full and respective PbE calculations with different threshold values. The largest threshold value of 1% corresponds to having the DPP core only in the active region of the PbE calculation. At 0.25% the carbon atoms up to and including the branching atom are included. For lower values, more and more side chain atoms are included. One can clearly see that the quantitative agreement between full and PbE calculations is indeed sensitive to the choice of the active region. No inclusion of any side chain atoms yields a –0.15 eV deviation for the HOMO energy on PbE-KS level already. The deviation is larger in PbE-*G*_0_*W*_0_ (−0.40 eV) for the HOMO, and we additionally find 0.24 eV for the LUMO, as well as −0.26 eV for the S_1_ energy. The data in Figure 2 confirms that lowering the threshold, and thereby adding more atoms to the active region, systematically improves the agreement between the results from full and PbE calculations. For instance, using a threshold value of 0.1% (addition of the four next carbon atoms) reduces the deviations to below 0.1 eV for all energies.

**Figure 3:**
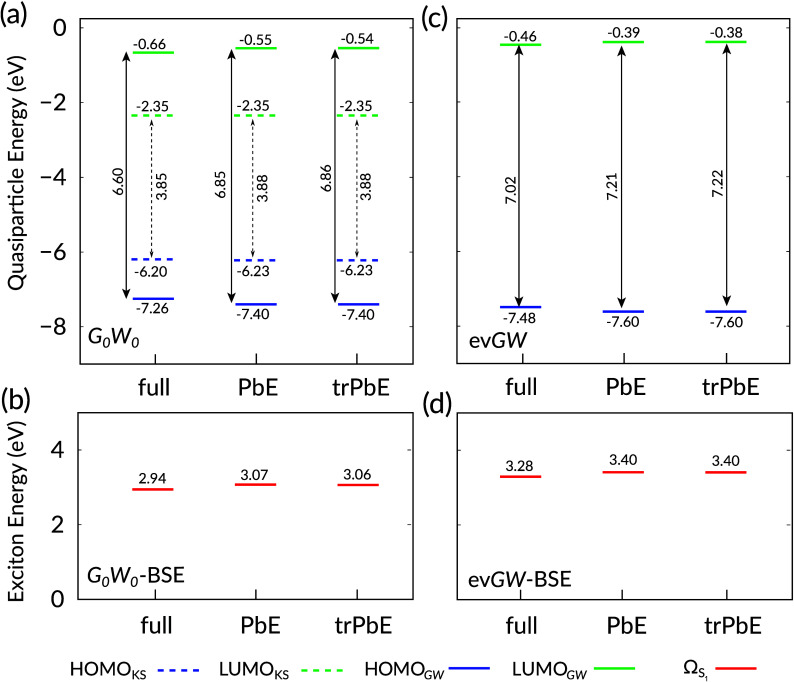
Near-gap excitation energies (in eV) for a DPP bicyclic ring with branched alkyl side-chains showing HOMO (blue), LUMO (green), and lowest electron–hole excitation (red) energies on KS level (dashed) or *GW*-BSE level (solid), as resulting from full, PbE, and trPBE calculations, respectively. Panels (a) and (b) show results from *G*_0_*W*_0_ calculations, panels (c) and (d) from ev*GW* calculations. Double-headed arrows additionally indicate the respective HOMO–LUMO gaps. See also Table 2 for details.

We will now inspect the different effects in the *GW*-BSE steps upon embedding without significant modification of the respective orbitals, using an active region to include the CH groups until the respective branching carbon atom for all following PbE calculations (corresponding to the threshold value of 0.25% above). Figure 3 shows the results of *G*_0_*W*_0_-BSE (panels (a) and (b)) and ev*GW*-BSE (panels (c) and (d)) calculations for the HOMO, LUMO, and Ω_S1_ energies for full, PbE, and trPbE calculations, respectively. The results are also collected in Table 2, in which additionally the individual contributions to the quasiparticle energies according to ε^QP^ = ε^KS^ – *V*^xc^ + Σ^x^+Σ^c^, the HOMO–LUMO gap, and contributions of the free transition energy (*D*), the exchange (*K*^x^) and direct (*K*^d^) terms of the electron–hole interaction to the electron–hole excitation energy Ω_S1_ are listed. The contribution *D*, *K*^x^ and *K*^d^ are calculated by forming the expectation value of the singlet BSE Hamiltonian with elements (eq 22) in the electron–hole wave function given by eq 14, and summing all resonant and antiresonant contributions arising from free-transitions, and the exchange and direct electron–hole interaction kernels, respectively. The quantity Δ_full_^PbE^ is the difference of the PbE calculation to the full one, and Δ_PbE_^trPbE^ measures the additional change of the basis set truncation with respect to the PbE calculation.

**Table 2:** Results of full-*G*_0_*W*_0_-BSE, PbE-*G*_0_*W*_0_-BSE, and trPbE-*G*_0_*W*_0_-BSE Calculations for a DPP Bicyclic Ring with Branched Alkyl Side-Chains^a^

		full	PbE	Δ_full_^PbE^	trPbE	Δ_PbE_^trPbE^
DFT	ε_H_^KS^	–6.20	–6.23	–0.03	–6.23	0.00
	ε_L_^KS^	–2.35	–2.35	0.00	–2.35	0.00
	*E*_gap_^KS^	3.85	3.88	0.03	3.88	0.00
*G*_0_*W*_0_	V_H_^xc^	–12.52	–12.47	0.05	–12.47	0.00
	Σ_H_^x^	–14.08	–14.02	0.06	–14.02	0.00
	Σ_H_^c^	0.50	0.38	–0.12	0.38	0.00
	*V*_L_^xc^	–11.25	–11.23	0.02	–11.23	0.00
	Σ_L_^x^	–7.52	–7.50	0.02	–7.50	0.00
	Σ_L_^c^	–2.04	–1.92	0.12	–1.92	0.00
	ε_H_^QP^	–7.26	–7.40	–0.14	–7.40	0.00
	ε_L_^QP^	–0.66	–0.55	0.11	–0.54	0.01
	*E*_gap_^QP^	6.60	6.85	0.25	6.86	0.01
BSE	*D*	7.63	8.00	0.37	8.00	0.00
	*K*^x^	0.54	0.56	0.02	0.56	0.00
	*K*^d^	–5.23	–5.49	–0.26	–5.50	–0.01
	Ω_S_1__	2.94	3.07	0.13	3.06	–0.01

a KS and QP HOMO and LUMO energies together with the individual contributions from the exchange-correlation potential *V*^xc^ and the self-energy split in Σ^x^ and Σ^c^, according to ε^QP^ = ε^KS^ – *V*^xc^ + Σ^x^ + Σ^c^, as well as KS and QP HOMO–LUMO gaps. The lowest electron–hole excitation energy from the respective BSE calculation Ω_*S*_1__ is also split into the free transition energy (*D*) and contributions from the exchange (*K*^x^) and direct (*K*^d^) terms of the electron–hole interaction. All energies in eV.

We first start the discussion of the KS HOMO and LUMO energies on DFT level only. They are shown in Figure 3(a) as dashed lines (blue: HOMO, green: LUMO) in the *G*_0_*W*_0_ panel (note that they are identical in the ev*GW* case). With the choice of the active region as discussed above, the change in the energy of the HOMO level upon embedding is small (−0.03 eV), and the LUMO is unaffected as expected from the theoretical basis given in Section 2.2. Using a truncated atomic orbital basis does not yield any changes of the KS electronic structure at the shown accuracy. As one can see from the values *N*_basis_ in Table 1, the basis truncation procedure as described in Section 2.3 removes only 42 of the 1194 functions, or about 3.5%.

Considering from now on the actual *G*_0_*W*_0_ results, one can first identify the typical effects of quasiparticle corrections on the HOMO and LUMO energies. In the full approach, the HOMO energy is lowered by 1.06 eV, and the LUMO energy is raised by 1.69 eV, such that the HOMO–LUMO gap increases by 2.75 eV. Upon PbE, however, the respective shifts are more pronounced, by 0.14 eV (0.11 eV) for the HOMO (LUMO), cf. Table 2. The quasiparticle gap *E*_gap_^QP^ is hence increased by 0.25 eV. Within the *GW* formalism, a larger gap can often be associated with reduced screening.^51,60,61^ This notion is corroborated by the data provided for the contributions from *V*^xc^, Σ^x^, and Σ^c^ to ε^QP^ in Table 2). From the respective Δ_full_^PbE^ for, e.g., the HOMO, one can see that the differences from the KS exchange-correlation potential and the exchange part of the self-energy almost compensate (note that *V*^xc^ is used with a negative sign in eq 3), and that consequently the differences in quasiparticle energies between full- and PbE-*G*_0_*W*_0_ are practically determined by effects in the correlation part Σ^c^ alone. For the LUMO, very similar observations can be made. Again, due to the minimal reduction of the basis set, no significant changes are noted in trPbE-*G*_0_*W*_0_ compared to PbE-*G*_0_*W*_0_.

Turning now toward the respective BSE results, we first note in the full calculation that the excitation energy results as 2.94 eV, which consists of the effective free-transition energy *D* = 7.63 eV, and the exchange (*K*^x^ = 0.54 eV) and direct (*K*^d^ = −5.23 eV) parts of the electron–hole interaction kernel. The fact that *D* exceeds *E*_gap_^QP^ by about one eV indicates that the electron–hole excitation is not exclusively given by a HOMO–LUMO transition. Qualitatively, the same holds also in the PbE (and trPbE) calculations. The S_1_ energy in PbE-*G*_0_*W*_0_-BSE results with 3.07 eV only 0.13 eV higher than in the full reference. This is noteworthy because as discussed above *E*_gap_^QP^ is larger by almost twice this value. Upon inspection of the individual contributions to the BSE level given in Table 2, one first observes that the free interlevel contribution *D* is larger by 0.37 eV and exceeds the relative increase on *E*_gap_^QP^, indicating that the lack of screening in Σ^c^ is larger for states outside the fundamental gap. That Ω_S1_ as obtained in the PbE approach is close compared to the full one despite these observations is due to the effect of the direct electron–hole interaction in the BSE Hamiltonian. This contribution, which is solely responsible for effective electron–hole binding, is with *K*^d^ = −5.49 eV in the PbE case stronger by 0.26 eV. As discussed in Section 2.1, the direct term contains the screened Coulomb interaction *W*. Lack of screening from the inactive region then implies that the electron–hole pair is subject to a stronger, more bare Coulomb like, electron–hole attraction. Such a stronger binding compensates at least to some extent (by ∼50%) the relatively larger energy differences of the free transitions.^62,63^

In Figure 3(c) and (d), the energy level diagram is given for results in which the ev*GW* method is used in all three cases. Generally ev*GW* leads here to a larger quasiparticle gap (by 0.42 eV) and a larger Ω_S1_ (by 0.34 eV) than in *G*_0_*W*_0_ (full approach). Interestingly, the difference in *E*_gap_^QP^ upon PbE is here with 0.13 eV somewhat smaller. Compared with a larger reference value in the full ev*GW* calculation, this reduces its relative error from 3.8% to 2.7%. For the first electron–hole excitation energy, the absolute deviation to the full calculation is with 0.12 eV almost identical to the *G*_0_*W*_0_ case. Due to the larger reference value, we still find a slightly reduced relative deviation of 3.7% compared to 4.4%. As before, basis truncation has no noticeable impact on the energy level diagram.

### 4.3 Prodan in Water

The study on the DPP bicyclic ring with branched alkyl side chains in the previous section has indicated that the proper choice of the active region is important, that differences in the energy levels between full and PbE calculations are attributable to the lack of screening effects from the inactive region, and that basis truncation had a minimal effect. We will now turn to a different test system, to scrutinize if these findings are specific to the DPP system in which the active and inactive regions were connected by a covalent bond. We will also present and analyze the use of PbE in the *GW*-BSE/MM scenario for quantum–quantum-classical embedding.

The system we have chosen for this study consists of prodan, a polarity-sensitive dye, solvated in bulk water, as shown in Figure 1(b). Up excitation of the S_1_ state absorption of a photon, the dipole moment of prodan in the excited state is significantly increased compared to the ground state. In polar solvents, such as water, additional screening effects originate from the structural relaxation of the solvent molecules, which in turn affect the excited state properties of the solute, and lead to a significant reduction of the emission energy. In ref (64), this process was simulated by an iterative *GW*-BSE+MD procedure. We take a single snapshot of one of those trajectories and perform static and polarizable *G*_0_*W*_0_-BSE/MM calculations on this structure.

To begin with, the system is partitioned in quantum and classical regions. We assign the prodan molecule and any water molecule whose center-of-mass is within 1 nm of the solute center-of-mass to the QM region. As a result, the QM region contains 199 atoms (prodan and 55 water molecules), which can be considerably challenging for full *GW*-BSE calculations. This QM region is then embedded in another 1 nm wide shell, which contains 630 water molecules treated on MM level.

**Figure 4:**
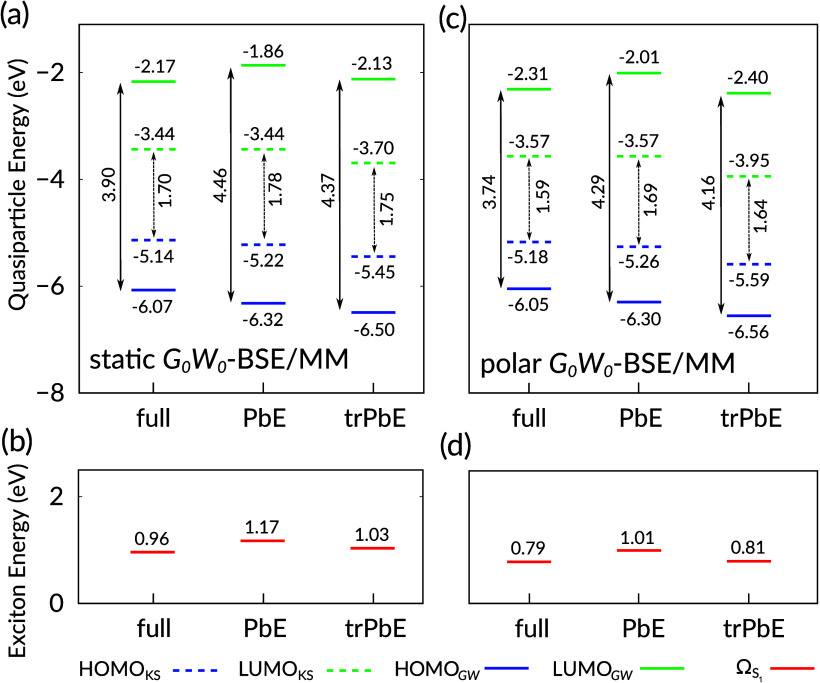
Near-gap excitation energies (in eV) diagram for aqueous prodan showing HOMO (blue), LUMO (green), and lowest electron–hole excitation (red) energies on KS level (dashed) or *GW*-BSE level (solid), as resulting from full, PbE, and trPBE calculations, respectively. Only the dye is included in the active region. Panels (a) and (b) show results from static *G*_0_*W*_0_-BSE/MM calculations, panels (c) and (d) from polar *G*_0_*W*_0_-BSE/MM calculations. Double-headed arrows additionally indicate the respective HOMO–LUMO gaps. See also Table 3 for details.

We begin with the results of full *G*_0_*W*_0_-BSE calculations in a static MM environment, as shown in Figure 4(a) and (b) and data summarized in Table 3. From the computational details in Table 1 it can be inferred that the computational cost for such a calculation is significant. We will discuss this in more detail in Section 5.1 below. In the *G*_0_*W*_0_ step, we observe the typical lowering of the HOMO energy and increase of the LUMO energy with respect to the KS reference, resulting in a HOMO–LUMO gap of *E*_gap_^QP^ = 3.90 eV as compared to *E*_gap_^KS^ = 1.70 eV. For the PbE calculations, we now first split the QM region into an active region containing only the dye molecule and an inactive region containing the 55 water molecules (setup indicated as ”dye” in Table 1 and Table 3). One can see from the dashed lines in Figure 4(a) that the PbE lowers the HOMO energy by 0.08 eV and yields a correspondingly increased HOMO–LUMO gap as the LUMO energy remains constant.

**Table 3:** Results of Full-*G*_0_*W*_0_-BSE/MM, PbE-*G*_0_*W*_0_-BSE/MM, and trPbE-*G*_0_*W*_0_-BSE/MM Calculations (All Static) for Aqueous Prodan^a^

			dye				dye + water	
		full	PbE	Δ_full_^PbE^	trPbE	Δ_PbE_^trPbE^	PbE	Δ_full_^PbE^
DFT	ε_H_^KS^	–5.14	–5.22	–0.08	–5.45	–0.23	–5.15	–0.01
	ε_L_^KS^	–3.44	–3.44	0.00	–3.70	–0.26	–3.44	0.00
	*E*_gap_^KS^	1.70	1.78	0.08	1.75	–0.03	1.71	0.01
*G*_0_*W*_0_	V_H_^xc^	–13.02	–12.79	0.23	–12.80	–0.01	–12.97	0.05
	Σ_H_^x^	–16.08	–15.86	0.22	–15.85	0.01	–16.04	0.04
	Σ_H_^c^	2.13	1.98	–0.15	2.00	0.02	2.03	–0.10
	*V*_L_^xc^	–11.31	–10.93	0.38	–10.96	–0.03	–11.08	0.23
	Σ_L_^x^	–7.61	–7.21	0.40	–7.27	–0.06	–7.37	0.24
	Σ_L_^c^	–2.44	–2.15	0.29	–2.12	0.03	–2.23	0.21
	ε_H_^QP^	–6.07	–6.32	–0.25	–6.50	–0.18	–6.19	–0.12
	ε_L_^QP^	–2.17	–1.86	–0.31	–2.13	–0.27	–1.96	0.21
	*E*_gap_^QP^	3.90	4.46	0.56	4.37	–0.09	4.23	0.33
BSE	*D*	4.14	4.76	0.62	4.71	–0.05	4.52	0.38
	*K*^x^	0.11	0.18	0.07	0.18	0.00	0.15	0.04
	*K*^d^	–3.30	–3.77	–0.47	–3.86	–0.09	–3.64	–0.34
	Ω_S_1__	0.96	1.17	0.21	1.03	–0.14	1.03	0.07

a KS and QP HOMO and LUMO energies together with the individual contributions from the exchange-correlation potential *V*^xc^ and the self-energy split in Σ^x^ and Σ^c^, according to ε^QP^ = ε^KS^ – *V*^xc^ + Σ^x^ + Σ^c^, as well as KS and QP HOMO–LUMO gaps. The lowest electron–hole excitation energy from the respective BSE calculation Ω_*S*_1__ is also split into the free transition energy (*D*) and contributions from the exchange (*K*^x^) and direct (*K*^d^) terms of the electron–hole interaction. All energies in eV.

Inspection of the Mulliken populations of the HOMO in the full KS calculation, as done in the previous section, reveals strong contributions from several water molecules close to the dye. With the restriction of the active region to prodan only, the neglect of these contributions changes the electronic state noticeably. Even though there is no covalent bond formed, this is very similar to the case of the DPP structure in the previous section, in which the HOMO extended over some carbon atoms of the side chain.

The quasiparticle gap in static PbE-*G*_0_*W*_0_-BSE/MM results with 4.46 eV considerably larger than in the full reference (3.90 eV), compatible with the earlier observations about reduced screening effects for the embedding. Indeed, the differences in the respective contributions from *V*^xc^ and Σ^x^ nearly compensate and the difference in quasiparticle corrections from full- to PbE-*G*_0_*W*_0_ mostly arises from effects in Σ^c^. Basis set truncation removes 541 basis functions (see Table 1) in aqueous prodan and has a more pronounced impact on the electronic levels. As can be seen in Figure 4 and Table 3, both HOMO and LUMO levels on KS level result lower in energy in trPbE as compared to the reference system (by 0.31 eV and 0.26 ev, respectively) and also to the respective PbE results. As a consequence of the almost constant shift, *E*_gap_^KS^ is only 0.05 eV larger. On *G*_0_*W*_0_ level, we note that the differences in the contributions in the quasiparticle corrections are relatively small and lead to only a small reduction of the quasiparticle gap by 0.09 eV. On BSE level, the calculated Ω_S1_ energy varies from 0.96 eV (full) via 1.17 eV (PbE) to 1.03 eV (trPbE). Inspection of the respective contributions to the BSE energy from the data in Table 3 reveals the same qualitative behavior as discussed in Section 4.2: the lack of screening from the inactive region increases the contribution from free transitions *D*, which is compensated to some extent by the for the same reason also increased electron–hole attraction in *K*^d^ (∼0.5 eV).

In the last two columns of Table 3, we additionally show results of PbE-*G*_0_*W*_0_-BSE results in which the six water molecules with Mulliken populations above 0.1% in the full system HOMO orbital are moved from the inactive to the active region. As can be seen, the change in the HOMO energy is very small compared to the full system, indicating that the more extended active region is a suitable choice. In this scenario, the differences in the quasiparticle energies for the HOMO and LUMO are smaller, but still amount to a *E*_gap_^QP^ increased by 0.33 eV. Different choices for the active/inactive region splitting do not affect the qualitative observation that this difference is effectively only given by Σ^c^ contributions. In the BSE calculation, the lowest electron–hole excitation energy is obtained as 1.03 eV, only 0.07 eV higher than in the full *G*_0_*W*_0_-BSE calculation. Here, the stronger by −0.34 eV electron–hole interaction in the BSE kernel compensates the larger by 0.38 eV free transition contribution.

When polarizable *GW*-BSE/MM is employed, the respective calculations of the coupled system in the self-consistent reaction field of the MM environment become state-dependent. What is shown as polar *G*_0_*W*_0_-BSE/MM in Figure 4(c) and (d) are the energy levels in the final step of a self-consistent procedure to evaluate the total energy of the S_1_ excited state according to eqs 34−35. Note also that the PbE and trPbE results shown here are for the case in which only the prodan molecule is in the active region, as in the left panel. We refrain from analyzing the shown data in detail because even though the exact numbers are different, there are no fundamental differences in what has been observed for static *G*_0_*W*_0_-BSE. In other words, while the external potential is different in both cases, the intrinsic effects going from full to PbE or trPbE calculations are the same.

### 4.4 Benzene-TCNE Dimer in Water

The final system under consideration is a dimer of benzene and TCNE, as it is known to exhibit intermolecular charge-transfer type excitations in which the hole is predominantly located on benzene and the electron on TCNE molecule.^65^ In a polar solvent, these CT excitations are massively lowered in energy compared to the vacuum case, and proper treatment of the polarizable environment is essential. Not only is the type of electron–hole excitation different from the ones studied in Section 4.2 and Section 4.3 but also the localization on the contributing frontier orbitals. We scrutinize in the following if the observations regarding PbE- or trPbE-*GW*-BSE calculations made for the previous two test systems also hold in case of intermolecular excitations. To this end, we prepared first a dimer of benzene and TCNE molecules stacked with a separation of 3.7 Å. This initial structure was then solvated with water using packmol.^66,67^ From this solvated system, the benzene-TCNE dimer and the 26 closest water molecules have been selected for the following calculations. Note that we are only interested in the trends of effects from using PbE or trPbE here, so a more involved procedure to obtain relaxed atomic positions is not required for this purpose.

**Figure 5:**
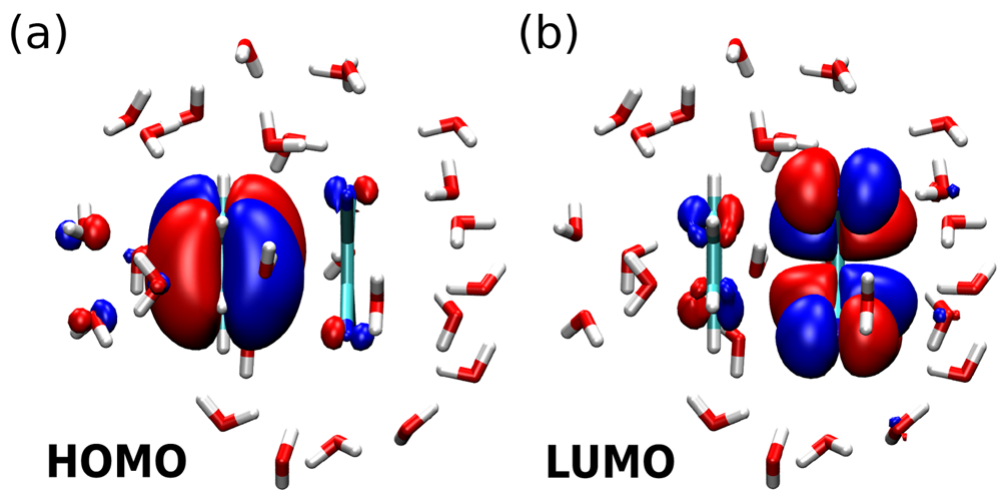
Isosurfaces of the (a) HOMO and (b) LUMO (isovalues ±0.01 a_B_^–3^) of the aqueous benzene-TCNE dimer as obtained from regular KS-DFT.

In Table 4 we show the resulting energy levels and their compositions for the different variants of *G*_0_*W*_0_-BSE calculations on this benzene-TCNE dimer, including a reference calculation for the dimer in vacuum. In the vacuum reference, the HOMO–LUMO gap is increased in *G*_0_*W*_0_ by 3.44 eV compared to the KS value, and the CT excitation energy is obtained as 3.10 eV. When embedded in water, the KS gap is reduced by 0.54 eV compared to the vacuum calculation, which is predominantly caused by a shift in the HOMO level. The bjoernfull-*G*_0_*W*_0_ gap is even reduced compared to vacuum by 0.81 eV, but interestingly here we also observe a downward shift of the LUMO energy albeit only by 0.19 eV compared to the upward shift by 0.62 eV of the HOMO. From the isosurfaces of the HOMO and LUMO in the full-*G*_0_*W*_0_ calculation as shown in Figure 5 one can clearly see the donor–acceptor character of the dimer in the distribution of the frontier orbitals on the respective molecules, corroborating the notion that a transition from HOMO to LUMO is of charge-transfer character. Its CT excitation energy is obtained as 2.41 eV, lower by 0.69 eV than in the vacuum case. It is noteworthy that this lowering of the CT energy is caused mainly by the reduction of the contribution from the free transition term *D* (6.41 eV in vacuum vs 5.59 eV in water) while the direct part of the electron–hole interaction is just reduced by 0.12 eV due to the additional screening from the environment.

**Table 4:** Results of Full-*G*_0_*W*_0_-BSE, PbE-*G*_0_*W*_0_-BSE, and trPbE-*G*_0_*W*_0_-BSE Calculations for a Benzene-TCNE Dimer in Water^a^

						10^–4^		10^–5^	
		vacuum	full	PbE	Δ_full_^PbE^	trPbE	Δ_PbE_^trPbE^	trPbE	Δ_PbE_^trPbE^
DFT	ε_H_^KS^	–7.79	–7.28	–7.29	–0.01	–7.20	0.09	–7.28	0.01
	ε_L_^KS^	–4.85	–4.88	–4.88	0.00	–5.02	–0.14	–4.87	0.01
	*E*_gap_^KS^	2.94	2.40	2.41	0.01	2.18	–0.23	2.41	0.00
*G*_0_*W*_0_	V_H_^xc^	–10.40	–10.46	–10.40	0.06	–10.43	–0.03	–10.39	0.01
	Σ_H_^x^	–11.97	–12.02	–11.95	0.07	–11.95	0.00	–11.95	0.00
	Σ_H_^c^	0.10	0.20	0.12	–0.08	0.11	–0.01	0.12	0.00
	*V*_L_^xc^	–11.54	–11.56	–11.52	0.04	–11.53	–0.01	–11.52	0.00
	Σ_L_^x^	–7.88	–7.93	–7.89	0.04	–7.92	–0.03	–7.89	0.00
	Σ_L_^c^	–1.68	–1.82	–1.69	0.13	–1.70	–0.01	–1.69	0.00
	ε_H_^QP^	–9.26	–8.64	–8.72	–0.08	–8.61	0.11	–8.72	0.00
	ε_L_^QP^	–2.88	–3.07	–2.94	0.13	–3.10	–0.16	–2.93	0.01
	*E*_gap_^QP^	6.38	5.57	5.78	0.21	5.51	–0.27	5.79	0.01
BSE	*D*	6.40	5.59	5.80	0.21	5.52	–0.28	5.80	0.00
	*K*^x^	0.01	0.01	0.01	0.00	0.01	0.00	0.01	0.00
	*K*^d^	–3.31	–3.18	–3.36	–0.18	–3.43	–0.07	–3.36	0.00
	Ω_S_1__	3.10	2.42	2.45	0.03	2.10	–0.35	2.45	0.00

a KS and QP HOMO and LUMO energies together with the individual contributions from the exchange-correlation potential *V*^xc^ and the self-energy split in Σ^x^ and Σ^c^, according to ε^QP^ = ε^KS^ – *V*^xc^ + Σ^x^ + Σ^c^, as well as KS and QP HOMO–LUMO gaps. The lowest electron–hole excitation energy from the respective BSE calculation Ω_*S*_1__ is also split into the free transition energy (*D*) and contributions from the exchange (*K*^x^) and direct (*K*^d^) terms of the electron–hole interaction. All energies in eV.

From Figure 5 it can also be seen that both frontier orbitals are not completely localized on the respective molecules, as the HOMO has minimal contributions at the TCNE and some close water molecules. The LUMO shows a similar pattern. Neverthless, for demonstration purposes and to assess whether the choice of an active region based on chemical intuition recovers the strong solvatochromic shift of the excitation energies, we choose only the dimer as the active region in the PbE and trPbE calculations. As one can see in Table 4, the associated neglect of the contributions of the water molecules to the HOMO has very little effect on the KS HOMO energy in PbE, and concomitantly, the KS HOMO–LUMO gap. The quasiparticle energies in PbE-*G*_0_*W*_0_ are 0.08 eV lower for the HOMO and 0.13 eV higher for the LUMO, compared to the full-*G*_0_*W*_0_ result, and as a consequence, the gap is larger by 0.21 eV. As observed for the DPP system and aqueous prodan, the differences in the respective contributions to the quasiparticle corrections from *V*^xc^ and Σ^x^ mostly cancel out. The CT excitation energy in PbE-*G*_0_*W*_0_-BSE is with 2.45 eV only 0.03 eV larger than in the full calculation, as the comparatively stronger electron–hole interaction almost completely compensates the larger free quasiparticle transition energies. Upon truncation of the basis set with the standard threshold of 10^–4^, we find unexpectedly larger deviations in trPbE-*G*_0_*W*_0_. Already on Kohn–Sham level, we find that the HOMO energy is increased to −7.20 eV and the LUMO lowered to 5.02 eV, leading to *E*_gap_ being reduced by 0.23 eV. At quasiparticle level, the gap energy is reduced by 0.27 eV as compared to the untruncated PbE case, so that the quasiparticle gap is very close to the full-*G*_0_*W*_0_ result. This should be considered coincidental. Indeed, the energy of the CT excitation is also lower by 0.35 eV and is as a consequence 0.32 eV smaller than in the full calculation reference, as the electron–hole interaction remains under-screened. Reducing the threshold value in the basis set truncation to 10^–5^ makes the trPbE-*G*_0_*W*_0_ results (see Table 4) agree with the not truncated case. However, in this case one only removes 12 basis functions, cf. Table 1. We therefore recommend to carefully scrutinize the truncation parameter in practical calculations on the KS reference frontier orbital energies before performing the actual *GW*-BSE steps.

## 5 Discussion

**Figure 6:**
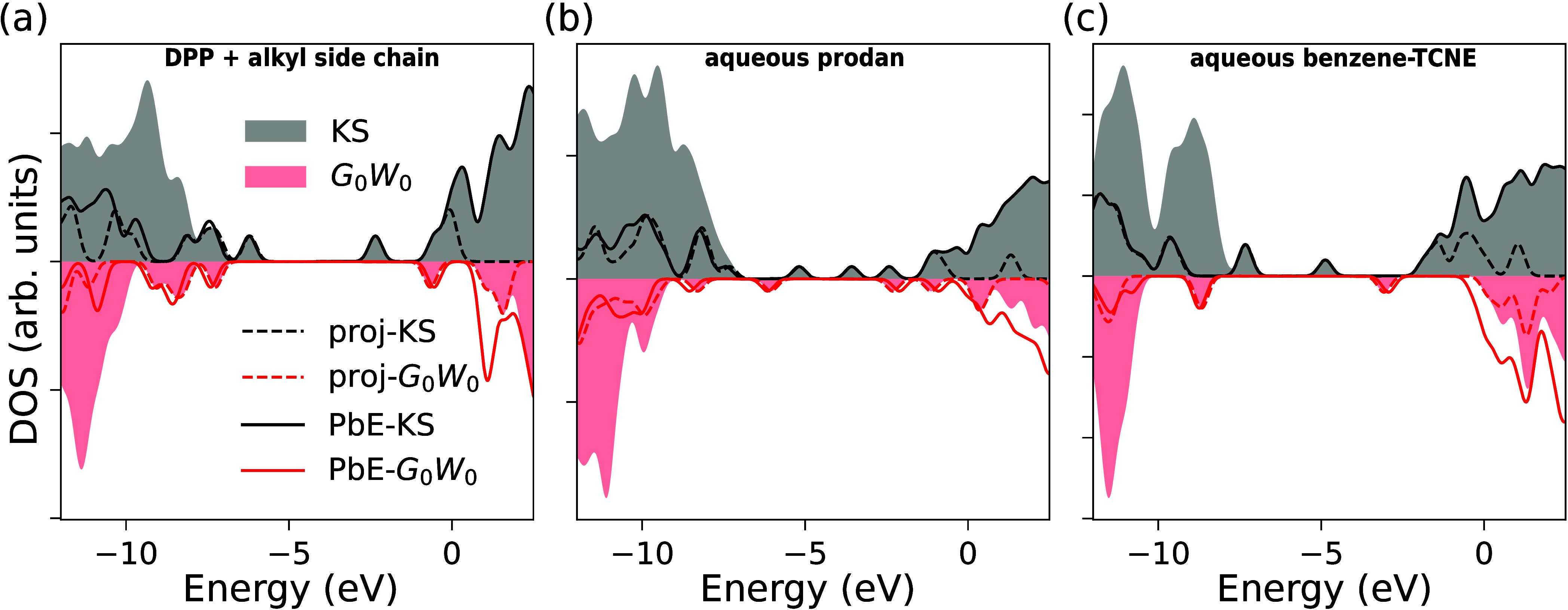
Density of states (DOS) for the three systems studied in this work: (a) DPP with branched alkyl side chains, (b) aqueous prodan, and (c) aqueous benzene-TCNE. Gray (red) shaded areas show the DOS as obtained by full KS (*G*_0_*W*_0_) calculations, while the black (red) lines indicate the respective PbE-KS (PbE-*G*_0_*W*_0_) DOS. Dashed lines indicate a partial DOS (PDOS) associated to the active atoms in the respective full calculations. A Gaussian broadening with standard deviation 0.2 eV is used in all cases.

We begin the discussion of the analysis of the PbE and trPbE techniques in application to the *GW*-BSE methodology by taking a broader view on the obtained quasiparticle energies than just the frontier orbital and fundamental gap energies. To this end, we show in Figure 6 a comparison between the density of states (DOS) for the three systems studied in this work as obtained from full and PbE calculations on KS and *G*_0_*W*_0_ levels. We also show by dashed lines a partial density of states (PDOS) based on the full calculation, in which we consider only those molecular orbitals whose Mulliken populations on the active atoms exceed 0.4, corresponding to the threshold value used in the PbE procedure for selecting the active MOs, cf. Section 2.2. On KS level (gray shaded area: full; dashed black: PDOS; black line: PbE) one can see in all three molecular systems the frontier orbital peaks are well reproduced, and by construction also the full range derived from unoccupied orbitals in PbE. For the energy region lower than the respective HOMO energy, one can generally observe the presence of fewer states, as expected. Especially for the two water-solvated systems a significant part of the full DOS is removed by the embedding.In systems (a) and (b), there is a good agreement between the PbE-DOS and the PDOS for the next group of peaks with slightly more deviations at ebergies below −10 eV. In aqueous benzene-TCNE, the agreement extends nearly over the full energy range shown, indicating that there overall little mixing of occupied electronic states of the dimer and the solvent molecules.

When comparing the same data obtained on *G*_0_*W*_0_ level of theory, one can see the small deviations of the frontier orbital energies in PbE-*G*_0_*W*_0_ with respect to the full calculation. For lower energy occupied and higher energy unoccupied levels, the comparison is not so straightforward. When one compares PbE-*G*_0_*W*_0_ to PbE-KS, one can see similarities in the broad shape of the DOS, but also that not all orbital energies experience the same quasiparticle corrections. This seems to affect the virtual DOS above the LUMO more significantly. As one can see in PbE-*G*_0_*W*_0_ results in Figure 6(a) and (c) in particular, there are peaks in the DOS below those in the full calculation DOS. This is an indication that the QP corrections for these levels, whose KS reference energy is the same in full- and PbE-KS, are less pronounced when an embedded calculation is performed. This is different from the observation that quasiparticle corrections are generally stronger for the frontier orbitals due to the lack of screening.

To elucidate we consider in more detail the differences between the PbE and full calculation split among the different contributions to the QP energies as done for HOMO and LUMO in Table 3, now for LUMO+1 and LUMO+2 from the final step in the polarizable *G*_0_*W*_0_-BSE/MM calculations. The results are summarized in Table 5. For the LUMO+1, we find qualitatively the same behavior as for the LUMO as discussed in Section 4.3: the contributions from *V*^xc^ and Σ^x^ nearly cancel out, and the too weak screening in PbE leads to a 0.23 eV higher quasiparticle energy as compared to the full calculation. For LUMO+2, the same does not hold: the difference in contributions from *V*^xc^ and Σ^x^ is significant and contributes with −0.86 eV to the difference in quasiparticle energies. The contribution from the difference Σ^x^ is with 0.65 eV positive, consistent with the argument of too weak screening. Its magnitude is however much bigger than for LUMO and LUMO+1. In total, we find a quasiparticle energy that is *lower* by 0.21 eV in PbE as compared to the full calculation.

**Table 5:** Results of Full-*G*_0_*W*_0_-BSE/MM and PbE-*G*_0_*W*_0_-BSE/MM Calculations (All Polar) for Aqueous Prodan^a^

	LUMO+1			LUMO+2		
	full	PbE	Δ_full_^PbE^	full	PbE	Δ_full_^PbE^
ε^KS^	–2.32	–2.32	0.00	–1.06	–1.06	0.00
*V*^xc^	–10.72	–10.47	0.25	–8.19	–5.91	2.29
Σ^x^	–6.76	–6.52	0.24	–4.53	–3.11	1.43
Σ^c^	–2.66	–1.58	0.24	–2.23	–1.58	0.65
ε^QP^	–1.02	–0.79	0.23	0.37	0.16	–0.21

a KS and QP LUMO+1 and LUMO+2 energies together with the individual contributions from the exchange-correlation potential *V*^xc^, and the self-energy split in Σ^x^ and Σ^c^, according to ε^QP^ = ε^KS^ – *V*^xc^ + Σ^x^ + Σ^c^. All energies in eV.

**Figure 7:**
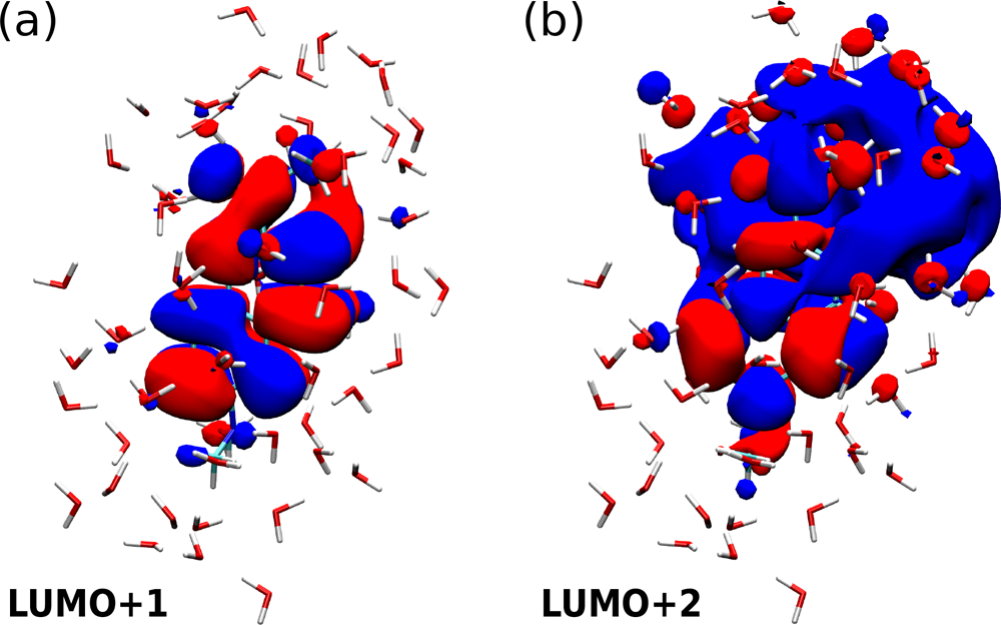
Isosurfaces of the (a) LUMO+1 and (b) LUMO+2 (isovalues ± 0.01 a_B_^–3^) of the aqueous prodan as obtained from regular KS-DFT.

Inspecting the nature of the respective states might provide an indication of the origin of this different behavior. In Figure 7 we show isosurfaces of LUMO+1 and LUMO+2, respectively. LUMO+1 (like LUMO) is predominantly localized on the dye molecule itself, with only small contributions from some close water molecules. In contrast, LUMO+2 is markedly different and extends to a large amount onto water molecules, that is into the inactive region from the point of view of projection-based-embedding. It stands to reason that in such a situation the removal of the occupied states of the inactive region from the expression for the exchange part of the self-energy introduces additional deviations.

### 5.1 Embedding Cost

**Figure 8:**
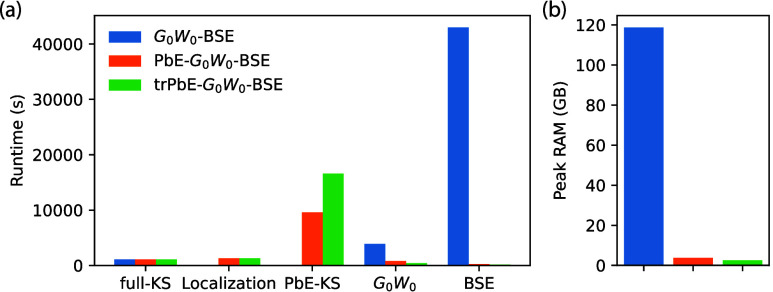
Computational costs of the different calculation steps in full-, PbE-, and trPbE-*G*_0_*W*_0_-BSE calculations for the aqueous prodan system from Section 4.3. (a) Runtime (in s) on 28 threads of an Intel(R) Xeon(R) Gold 5120 CPU @ 2.20 GHz. (b) Peak RAM consumption in GB.

In addition to the quality of the results of the different PbE-*G*_0_*W*_0_-BSE calculations compared to the full calculations, we consider the respective computational costs using the aqueous prodan system from Section 4.3 as an example. As can be seen from Table 1, large savings can be expected in the RPA steps (reduction of the number of transitions to less than 20%), and in the BSE solution (reduction of the product basis size to about 3%). Figure 8(a) shows the runtime of the respective calculation steps. For all three variants, the underlying DFT calculation on the full system is performed with ORCA in about 1100 s. Localizing all 336 occupied orbitals with the unitary optimization requires in VOTCA-XTP around 1300 s, and performing the PbE-KS around 9600 s. Note that the internal DFT implementation in VOTCA-XTP is intended only for development and testing purposes without performance optimizations. Therefore, the absolute timings for the PbE-KS step appear much larger relative to the full-KS calculation performed with ORCA. In principle, PbE-KS has the same scaling as regular KS. As expected, the most significant saving in computational time is the actual *G*_0_*W*_0_ and BSE steps of the procedure. For the former, the reduced number of transitions in the RPA, combined with a lower number of states for which quasiparticle corrections have to be determined, reduced the time from 3900 s to around 800 s. Note that the absolute cost of the *G*_0_*W*_0_ step is, in fact, reasonably small (only a factor ∼3.5 in the full calculation) compared to the DFT step, due to the use of the Plasmon-Pole Model for the frequency-dependence of the self-energy, which requires the explicit evaluation of the microscopic dielectric function only for two frequencies, see also Section 2.1. The most dramatic compute time reduction is observed for the BSE step. Here PbE leads to a reduction from 43000 s to just 270 s due to the massively reduced dimension of the product basis. Basis set truncation allows for another reduction of runtimes in *G*_0_*W*_0_ and BSE by a factor of 2, respectively.

While the timings are an important consideration in performing *GW*-BSE calculations, the peak memory consumption is in many case the more relevant bottleneck in limiting the accessible system sizes. From the data shown in Figure 8(b), it is clear that the full and PbE approaches differ vastly in peak memory consumption. Embedding and the subsequent reduction in the in-memory storage of three-center Coulomb integrals after contraction with molecular orbitals requires only 3.8 GB of RAM compared to 118.7 GB. The truncated basis has a smaller effect on top of this (2.5 GB). This clearly shows that PbE techniques can remove some computational bottlenecks of *GW*-BSE calculations, at the price of some deviations in the obtained results due to the lack of screening contributions from the inactive region.

## 6 Summary

In this paper, we have introduced and scrutinized projection-based-embedding techniques of *GW*-BSE calculations. Based on the analysis of the three test systems DPP ring with branched alkyl side chains, aqueous prodan, and an aqueous benzene-TCNE dimer, we could see that PbE can offer significant computational gains, making larger systems accessible to the many-body Green’s functions based methodology. We have demonstrated that it can also be directly incorporated in quantum-classical embedding (*GW*-BSE/MM) schemes. We also found that the agreement with full calculations depends on the choice of the active region and is subject to effects from the neglect of screening contributions from the inactive electrons in the *GW* steps, which leads generally to an increased quasiparticle HOMO–LUMO gap. It was also noted that the lack of screening is in part compensated in the BSE as it manifests itself in an increased electron–hole attraction so that deviations from full results for the electron–hole excitation energy are on the order of 0.1 eV for the different types of excitations studied here. It should be noted that all excitations are near-gap excitations, and it can not be guaranteed that the same quantitative agreement will hold for higher-energy excitations. Finally, we have seen that additional truncation of the basis set can reduce the computational costs by a factor of 2 with respect to full-basis PbE, but results appear to be sensitive to the chosen threshold values for removing basis functions.
